# A Visual Discrimination of Existing States of Virus Capsid Protein by a Giant Molybdate Cluster

**DOI:** 10.3390/nano12050736

**Published:** 2022-02-22

**Authors:** Yarong Xue, Mingfen Wei, Dingyi Fu, Yuqing Wu, Bo Sun, Xianghui Yu, Lixin Wu

**Affiliations:** 1 State Key Laboratory of Supramolecular Structure and Materials, Institute of Theoretical Chemistry, Jilin University, No.2699 Qianjin Street, Changchun 130012, China; xueyr17@mails.jlu.edu.cn (Y.X.); weimf19@mails.jlu.edu.cn (M.W.); 2 School of Pharmacy, Nantong University, No. 19 Qixiu Road, Nantong 226001, China; fudingyi@ntu.edu.cn; 3 State Engineering Laboratory of AIDS Vaccine, Jilin University, No.2699 Qianjin Street, Changchun 130012, China; bo_sun@jlu.edu.cn (B.S.); xianghui@jlu.edu.cn (X.Y.)

**Keywords:** colorimetric discrimination, giant polyoxometalate, HPV capsid protein, hypochromic of molybdate clusters, opposite color response

## Abstract

We report a unique phenomenon, the opposite color response of a giant polyoxometalate, (NH_4_)_42_[Mo_132_O_372_(CHCOO)_30_] (H_2_O)_72_ ([Mo_132_]), to the existing states of human papillomavirus (HPV) major capsid protein, L1-pentamer (L1-p), and virus-like particles (VLPs). The color responses originate from the different assembly forms between [Mo_132_] and the capsid protein. The latter were inspected and separated by using CsCl gradient centrifugation, and validated in detail by sodium dodecyl sulfate-polyacrylamide gel-electrophoresis (SDS-PAGE), dynamic light scattering (DLS), and transmission electron microscopy (TEM) imaging. Furthermore, the intrinsic mechanisms were investigated in-depth by using XPS-based semi-quantitative analysis and well-designed peptides, revealing the critical points of L1 that determine the charge–transfer ratio between Mo(V) to Mo(VI), and consequently, the levels of [Mo_132_] hypochromic in different assemblies. Such a unique phenomenon is significant as it supplies a colorimetry approach to distinguish the existing states of the HPV capsid protein and would be significant in the quality assay of the HPV vaccine and existing states of other viruses in the future.

## 1. Introduction

Human papillomavirus (HPVs) causes commonly transmitted infections that occur in humans [1,2]. Some types of HPV lead to severe diseases including a series of verrucas and cancers [3]. To effectively prevent the occurrence of infections, vaccines that specifically target the virus-induced diseases have been developed recently, and many efforts have focused on building a security shielding system [4] Virus-like particles (VLPs), formed from the self-assembly of pentamer subunits comprising major capsid protein L1 without the participation of DNA, have been demonstrated to be an essential resource of vaccines because of the similar surface structure and antigenic epitopes to those of actual viruses. As a kind of prophylactic vaccine, VLPs were shown to be effective for protecting several subtypes of HPV from infections [5,6]. On one hand, these assembled particulate structures are also resilient to most environmental stresses and are promising for eliciting an efficient and potent immune response [7]. On the other hand, during the development of prophylactic vaccines against high-risk types of HPVs, VLPs can also be adopted directly in mimicking more closely to the neutralized epitopes, morphology, and keeping the size of native ones [8]. However, the production and usage of spherical capsids as vaccines relies significantly on the VLP’s integrity, and any disassembly may result in losing effectiveness in producing antibodies. Therefore, the quick and convenient detection for the existing state of VLPs or their disassembly is crucial. Since the normal subunits of VLPs are the pentamer of capsid protein L1(L1-p), the sensitive determination of the species can demonstrate the incomplete state of VLPs [9]. Spectroscopic, dynamic light scattering, and electron microscopic measurements are commonly used characterization techniques [10,11], but they are not suitable for in situ quality assays during the preparation of vaccines. Therefore, it is imperative to develop a rapid and straightforward method for differentiating complete capsids and dissembled pentamers via a quick and direct procedure.

Polyoxometalates (POMs) are a kind of negatively charged inorganic clusters comprising early transition metal oxides [12]. The uniform shape and nanoscale size of POMs provides useful functional properties that can be useful in several emerging technologies including medicine [13,14]. Typically, the reduced coordination metal ions allow the deep-color clusters to have extended light absorption in the visible region. In the presence of oxidants, the reduced clusters exhibit reversible oxidation properties, shifting absorption toward another state [15]. Among the POM family, the giant molybdate clusters realized by Müller’s group exhibited an elegant architecture and morphology with larger sizes of more than 2.5 nm [16]. As giant clusters, which have been extensively studied including, for example, self-assemblies into nanoscale capsules and forming co-assemblies together with cationic organic components via electrostatic interactions [17], and their functionalization was either performed on biomedical applications [18]. We have reported the electrostatic interaction of the giant clusters with cationic amphiphiles for synergistic self-assembly, the behavior with biomolecules in solutions in adhesion, anti-bacterial properties as well as bio-imaging [19,20]. However, up until now, there have been no reports concerning the use of these POMs and other inorganic clusters on the detection of the existing state of virus capsids, either in the assembly or disassembly state, neither the ionic binding position with capsid proteins.

**Scheme 1:**
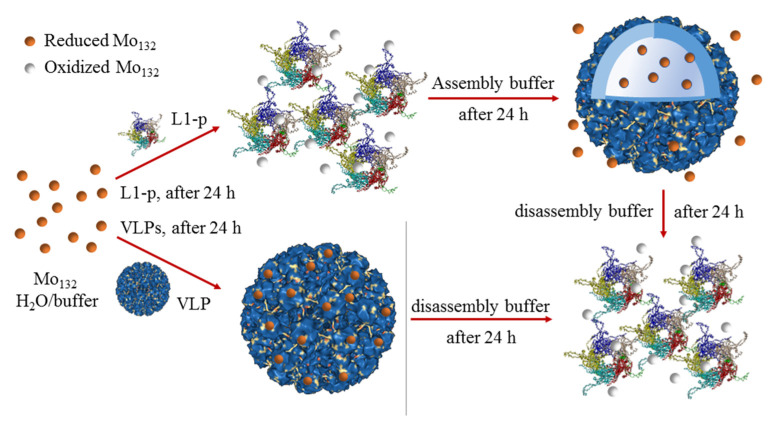
Schematic illustration of the binding modes and different color responses of [Mo_132_] to HPV L1-p and VLPs, respectively.

The Mo(V) have been shown to be essential components in giant molybdate POMs, making them show colors in solid and solution due to their absorption, corresponding to the intervalence charge transfer (IVCT) between Mo(V) and Mo(VI) [21,22]. Thus, the valence state change of giant clusters always yields a color change, and as a result, Mo(V) can be oxidized into Mo(VI), accompanied by a color fading of brick-red in the presence of oxidants. Furthermore, the color changes are reversible because the Mo(VI) at the highest oxidized state are reduced while the cluster structure is maintained, just like the oxidation at a reduced state [23,24]. These properties of POMs and their charged surface provide an excellent opportunity to detect the existing state of the proteins comprising capsids through the electrostatic interaction with free N-terminals bearing redox features. Thus, the states of some proteins that can be oxidized and reduced can be characterized through the color fading of giant POMs after the redox process. In this context, we herein selected a giant brown Keplerate cluster, (NH_4_)_42_[Mo_132_O_372_(CHCOO)_30_](H_2_O)_72_, abbreviated as [Mo_132_], which contains 72 Mo(VI) and 60 Mo(V), to sensitize the status of L1 proteins and the stability of the capsid [25]. Because L1 proteins at the states of assembly and disassembly show different binding modes with the [Mo_132_] cluster through exposing and shielding amino residues, we successfully realized the vital identification for the capsid proteins through simple color changes of [Mo_132_] (Scheme 1). The present method demonstrated a practical approach to examine the quality of the prophylactic HPV vaccines. Of course, the obtained results are also significant in widening bio-applications such as qualitative screening of HPV infections and tracking the differentiation process of HPVs in the human body by detecting L1 proteins.

## 2. Results and Discussion

### 2.1. Hypochromic Response of [Mo_132_] to L1-p

**Figure 1:**
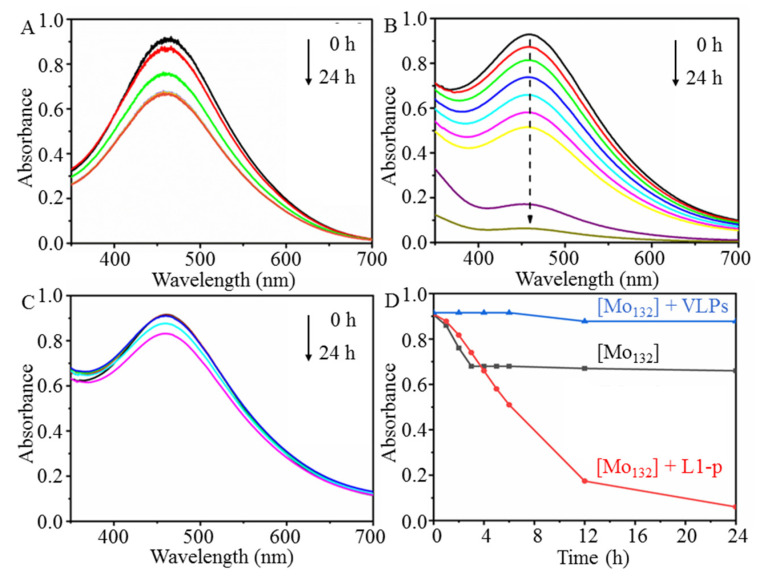
Time-dependent UV–Vis absorption spectra of [Mo_132_] (2.5 µM) in (**A**) buffer A, in the presence of (**B**) L1-p (10.0 µM) and (**C**) VLPs (10.0 µM), and (**D**) plots of the corresponding absorbance at 456 nm in (**A**–**C**) versus time for 24 h.

The [Mo_132_] (2.5µM) in buffer A shows a typical absorption band at 456 nm similar to the cluster in aqueous solution (Figure 1A), which can be assigned to the intervalence charge transfer (IVCT) between Mo(V) and Mo(VI) centers bridging by O atoms [21,22]. Since the absorption bands of the L1 protein at both states of pentamer and VLP appeared at wavelengths less than 300 nm, the changes in absorption spectra of the [Mo_132_] mixture with L1-p in the visible region was attributed to a source from the inorganic cluster. A gradual hypochromic behavior of [Mo_132_] in buffer A solution with time exposure in air was observed, and nearly 20% absorbance was lost after 4 h, when a turning point occurred. After that, no further change occurred for the absorption with time over one day. The hypochromic properties of [Mo_132_] can be attributed to the oxidation of partial Mo(V) in the giant cluster by oxygen in air, according to the opposite observation during its formation [24]. As further confirmed in the characterization, this color change in the aerobic environment does not affect the structural integrity and the detection for proteins because of the large number of reduced Mo(V) contained in one cluster.

In comparison to [Mo_132_] in buffer A, a much stronger hypochromic effect of the cluster occurred in the same solution along with the addition of L1-p. A linear decrease in absorbance was observed, and more than 80% of the original absorbance disappeared until cultured to a turning point at 12 h. Further color fading proceeded continuously, and more than 90% of absorbance vanished after 24 h (Figure 1B). Interestingly, when [Mo_132_] was added into the solution containing VLPs, only a little color degradation occurred over time (Figure 1C), and over 95% absorbance of the inorganic cluster was maintained at the time scale. The job plots of the [Mo_132_] cluster at the states in the presence of protein L1-p or VLPs show the apparent differences in the absorbance at the visible region versus the time (Figure 1D).

**Figure 2:**
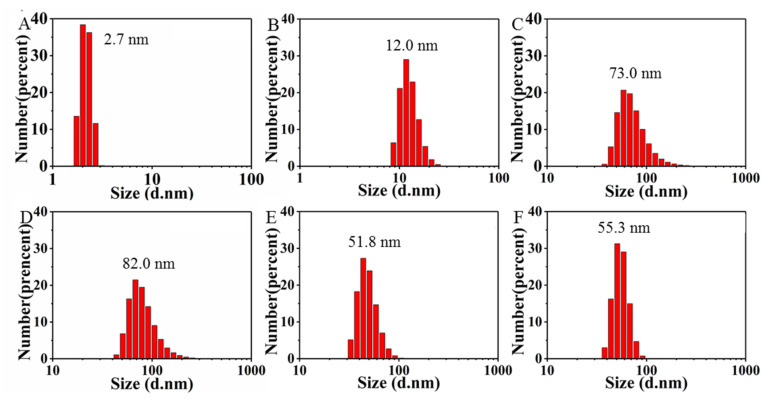
DLS histograms of (**A**) [Mo_132_] and (**B**) L1-p subunit in buffer A; the mixture of [Mo_132_] and L1-p (**C**) right after, and (**D**) after 24 h of incubation in buffer A; (**E**) the mixture of [Mo_132_] and L1-p after 24 h dialysis in assembly buffer; and (**F**) that of [Mo_132_] and the as-assembled VLPs in assembly buffer, respectively.

[Mo_132_] has been demonstrated to form blackberry-like hollow spherical self-assembled shapes in several tens of nanometers in aqueous solution [26] while maintaining a mono-dispersed state for a couple of days. As shown in Figure 2A, the DLS curve of the cluster alone showed a hydrodynamic diameter of 2.7 nm, similar to the size (2.9 nm) calculated from the crystal structure, illustrating the mono-dispersion and structural completeness [27,28]. The L1-p in buffer A had a size of about 12.0 nm (Figure 2B), being very close to the well-dispersed state of the pentamer subunit reported previously [29,30]. When the inorganic cluster was added into a solution containing L1-p, larger aggregates with a diameter of about 73 nm (Figure 2C) formed immediately. This size differs from each component or typical VLPs, implying the quick interaction between [Mo_132_] and the protein. The incubation for 24 h or longer did not lead to precipitation, but maintained a stabilized aggregate with a slight increase in size to 82 nm (Figure 2D). As already shown, the negatively charged POMs bind with some peptides containing basic residues through electrostatic interaction and hydrogen bonding [31,32]. The non-specific interaction can also induce the combination of the [Mo_132_] cluster bearing 42 negative charges with L1-p, causing the formation of larger assemblies or aggregates.

### 2.2. Visual Response of [Mo_132_] to the Isolated L1-p and VLPs

**Figure 3:**
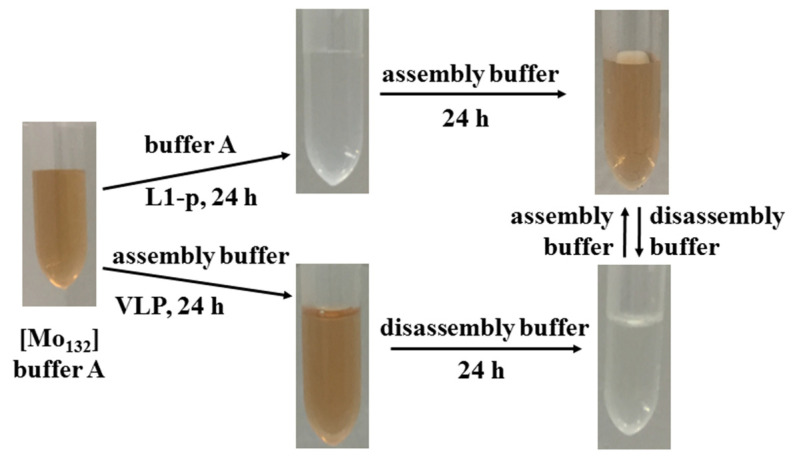
Color responses of [Mo_132_] in buffer A in the absence and presence of either L1-pentamer or the VLPs, respectively.

The absorption spectral changes of [Mo_132_] to L1-p and its VLP assembly (Figure S1) were examined in parallel with visual inspection through color observation. As shown in Figure 3, [Mo_132_] is sensitive in detecting the existing states of protein L1. In a normal process, the L1 monomer is very unstable and exists typically in a pentamer state, which further self-assembles into an empty capsid, VLP, under high ionic concentration and low pH, spontaneously [27,28]. To identify the possible influence of the [Mo_132_] cluster on the assembly process, the L1-p in a large amount of assembly buffer is monitored to allow for the assembly of VLP in the presence of [Mo_132_], following a published standard process for other POM [27]. After encountering a dialysis procedure, the DLS assay revealed the formation of characteristic assemblies accompanied by a size change from 12 nm at the beginning to an average of 51.8 nm after 24 h of incubation (Figure 2E), in perfect agreement with the full-sized scale (50–55 nm) of VLPs comprising of the HPV16 L1 protein [29,30]. This result indicates that the presence of [Mo_132_] does not affect the self-assembly of L1-p into VLP. However, after this assembly process, the dynamic small size attributed to [Mo_132_] was no longer observed, even after 24 h of incubation (Figure 2F), implying that most of the clusters were trapped either inside or on the outside surfaces of the formed VLPs, or were eliminated through dialysis. The existence of [Mo_132_] did not affect the further assembly of the L1-p as a subunit. Importantly, accompanying the self-assembly of L1-p, the faded color of [Mo_132_] recovered because the solution changed from colorless back to brown, the original color of the [Mo_132_] in solution (Figure 3).

### 2.3. Prevention of [Mo_132_] Hypochromic by VLPs

More interestingly, the addition of [Mo_132_] into an assembly buffer containing VLPs did not result in an obvious hypochromic after 24 h of incubation in the air; in contrast to L1-p, it showed much less color fading compared to a single cluster in buffer A, indicating the strong inhibition of VLPs on the color bleaching of [Mo_132_]. Meanwhile, the DLS histogram (Figure 2F) confirmed a well-kept full-size of VLPs in 24 h. To identify whether [Mo_132_] binds to VLPs or just remains isolated in solution, the ultracentrifugation of various components in gradient CsCl solution, were performed in tubes based on the principle of size and density dependence on the position to the rotating center, where components with the larger size and higher density will be located closer to the bottom.

**Figure 4:**
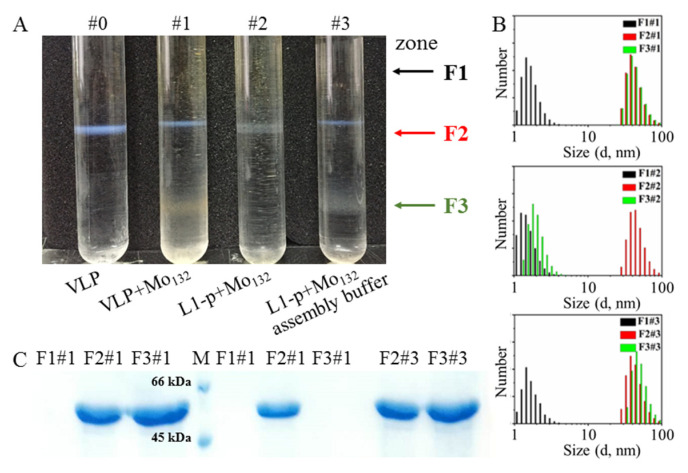
(**A**) The photographs after CsCl gradient ultracentrifugation in tubes for VLPs (tube #0), VLPs@[Mo_132_] in assembly buffer (tube #1); the mixture of L1-p and [Mo_132_] before (tube #2) and after (tube #3) monitoring in assembly buffer for 24 h, respectively. (**B**) DLS histograms of VLPs@[Mo_132_] (upper), the mixture of L1-p and [Mo_132_] before (middle) and after monitoring in assembly buffer (bottom), extracted from the position of zone F1 (black), F2 (red), and F3 (green), respectively. (**C**) SDS-PAGE of the corresponding component extracted from the position of F1#1, F2#1 and #3, and F3#1 and #3, respectively. The channel of M is the marker from the standard protein.

After the CsCl gradient ultracentrifugation, the photograph of VLP alone in tube #0 showed a single blue band because of the concentrated protein, ascribed to the position of complete VLPs, at zone F2 (Figure 4A) in the middle of the centrifugation tube. In the case of VLP mixing with [Mo_132_] in tube #1, besides a narrower blue belt at the F2#1, a wideband emerges at the lower position F3#1, suggesting the formation of aggregates with larger size differing from VLP or L1-p. Because the observed color of the belt near F3#1 was far from that of VLP at F2#1 while the sole VLP did not show any significant hint at the F3#0 position, we suggest that the [Mo_132_] traps VLPs to form larger-sized aggregates. As a result, the inhibition to the hypochromic effect of [Mo_132_] can be explained to be derived from protecting the VLPs from external oxidation. On the other hand, the photograph of L1-p mixing with [Mo_132_] in tube #2 presented a weak belt with a pale color at the position F2#2, similar to that of VLP at F2#0, but there was no obvious belt emerging at zone F3#2. The result implies that part of L1-p self-assembled into VLPs automatically in the presence of inorganic clusters, but almost no formation of the proposed aggregates comprising the formed VLPs, and [Mo_132_] was shown as the observation at zone F3#1. When L1-p was mixed with [Mo_132_] in the assembly buffer, as seen in tube #3, both narrow blue belts at zone F2#3 and wide yellowish-brown belts emerged simultaneously at F3#3. The identical phenomenon of F3#3 to F3#1 demonstrates the formation of VLP in the presence of an inorganic cluster and the induced aggregation. Looking at these results together, we can draw the following conclusions: (1) the color degradation and the possible interaction with clusters did not affect the assembly of L1-p; and (2) a cluster induced aggregation of VLPs occurred, which is consistent with the DLS measurements (Figure 2C,D).

The belts at different zones in tubes after CsCl gradient ultracentrifugation were taken out, and the corresponding protein was assayed via the SDS-PAGE technique (Figure 4C). Taking two known proteins with molecular weights of 44 and 66 kDa as markers, all products extracted from F2 zones of tube#1–#3 point to a molecular weight of ~53 kDa, which is perfectly consistent with the L1-monomer. Besides emerging in the zone F2 in all tubes, the L1 protein also appeared at zone F3 in tubes #1 and #3, supporting the assignment for VLP aggregation with [Mo_132_] at two zones. Meanwhile, the fact that no smaller proteins were observed in the F1 zones of tubes #2 and #3 shows that all the L1-p have already self-assembled into VLPs, especially in tube#2. Coincidently, the DLS histograms of the belts taken from tube #1 showed distributions of particle size that were consistent with [Mo_132_] at zone F1#1, and the particle sizes corresponding to that of the VLPs at zones F2#1 and F3#1 were determined (Figure 4C, upper). As the cluster was proposed to be trapped in VLP aggregates, no isolated [Mo_132_] was found at zone F3#1 due to the strong interaction between two components. For the belts extracted from tube #2 (Figure 4C, middle), besides identifying the particle that can be ascribed to [Mo_132_] at F1#2, we also observed the particle size attributed to VLPs at F2#2. Although a small size close to [Mo_132_] appeared at F3#2, no larger particles corresponding to the induced VLPs were observed excluding the possibility of it being there for [Mo_132_]. Again, the DLS histogram of tube #3 (Figure 4C, bottom) shows the full spectra of size distributions similar to [Mo_132_] at zone F1#3, VLPs at zone F2#3, and the cluster-triggered VLPs aggregation at zone F3#3, which were identical to the case observed in tube #1.

**Figure 5:**
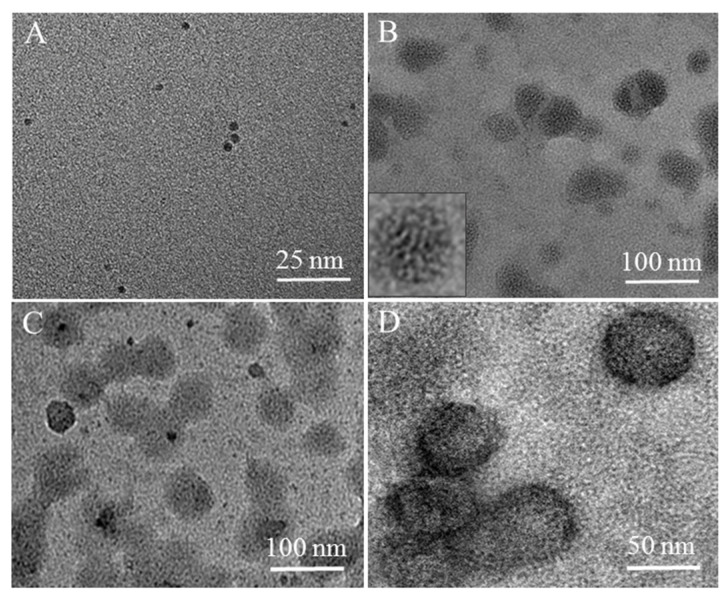
TEM images of (**A**) [Mo_132_] in buffer A; (**B**) the mixture of [Mo_132_] and L1-p in buffer A; (**C**) after 24 h dialysis in assembly buffer, and (**D**) the mixture of [Mo_132_] and the as-prepared VLPs in assembly buffer, respectively. All images were acquired directly on samples without the phosphotungstate stain.

To further verify the morphology and completeness of the formed VLPs taken from F2#1 and F2#3, TEM images of [Mo_132_] in buffer A, the mixture of [Mo_132_] with L1-p before and after assembly monitoring, and the mixture of [Mo_132_] and the as-prepared VLP were acquired. Because of the tiny size and mono-dispersion, the inorganic cluster (Figure 5A) could be well discerned from that of L1-p or VLPs in solution (Figure 5C,D). In the mixture of L1-p and [Mo_132_], whether assembly monitoring was performed or not, we consistently obtained spherical particles (Figure 5B,C) that can be ascribed to the formation of VLPs as they matched the size of VLP cavity [29,30]. The amplified images (inset of Figure 5B,C) show small particles attributed to the inorganic clusters located inside the inner wall. Therefore, Figure 5B provides additional evidence for the cluster-triggered assembly of VLPs from L1-p, even without assembly monitoring. In addition, partial inorganic clusters were observed encapsulated inside the VLPs during the triggered co-assembly with L1-p, indicating a strong interaction between [Mo_132_] and L1-p. The observation of VLPs can further support this analysis after mixing with [Mo_132_] (Figure 5D), where the inorganic clusters are mainly located at the outside surface of VLPs being very different from that of the co-assembly of the two components.

**Figure 6:**
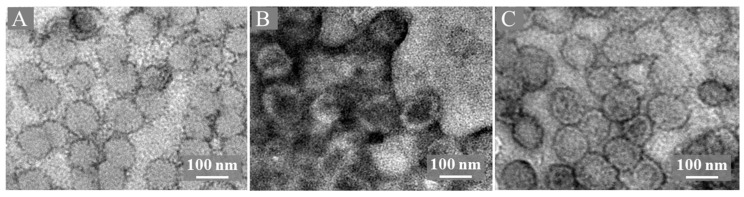
TEM images of (**A**) VLPs; (**B**) the mixture of [Mo_132_] and L1-p after 24 h dialysis in assembly buffer; (**C**) the mixture of [Mo_132_] and the as-prepared VLPs in assembly buffer, respectively. All of were extracted from the F2 fraction in tubes after CsCl gradient ultracentrifugation and stained with 2% phosphotungstate for 2 min before measurement.

To obtain the essential profile, the TEM images for the samples extracted from zones F2#0, F2#1, and F2#3 were then acquired with negative staining (Figure 6). The micrographs show that the topography of VLPs and the particle size become more uniform and precise. Besides the transparent empty shell (Figure 6A), the mono-disperse particles with a statistical diameter of about 55 nm (Figure 6B,C) were in good agreement with the reported full-size of VLPs [29,30]. From the foregoing, we concluded that [Mo_132_] could quickly bind with L1-p to form irregular aggregates, finally leading to the formation of VLP containing [Mo_132_] inside. However, once [Mo_132_] is mixed with the as-prepared VLPs, it assembles into [Mo_132_]@VLP, where the cluster particles bind to the surface of VLPs (Figure 6C). As the molecular weight of [Mo_132_] is much less than that of VLP, the sedimentation coefficient of [Mo_132_]@VLPs is close to that of the VLPs, thus the encapsulation of a few [Mo_132_] clusters does not significantly alter the surface properties of VLPs. Therefore, it is rational to explain that [Mo_132_]@VLPs display a close level to VLPs in tubes after the CsCl gradient ultracentrifugation (Figure 4A). The two adjoining lancet belts at the position of F2#2 suggest an appreciable difference between VLPs and those encapsulated with [Mo_132_]. Moreover, whether stained or not, the larger aggregates of VLPs induced by [Mo_132_] were clearly discernible in Figure 5 and Figure 6, which supports the observation of proteins in F3#1 and F3#3 (Figure 4B).

### 2.4. Colorimetry Response Mechanism of Mo_132_ to L1-p and VLP

**Redox nature of [Mo_132_].** Coordination atoms such as Mo and W of POMs at the highest oxidation state are known to be photochemically reduced in the presence of a reductant, and the reduced POMs showed oxidation properties such as peroxidase for some organic and bio-molecules [33,34]. [Mo_132_] has 60 reduced Mo(V) atoms and 72 oxidized Mo(VI) atoms, allowing the cluster to be both reduced and oxidized in suitable conditions. As the intervalent charge transfer absorption of Mo(V) to Mo(VI) emerged in the visible region, the [Mo_132_] normally appeared as yellowish-brown. However, when Mo(V) atoms are oxidized, color degeneration will occur. For example, its incubation with a weak reductant L-ascorbic acid (Vc) for 24 h does not change the [Mo_132_] cluster’s color through reduction (Figure S2A). However, instead of the phenomenon displayed in Figure 1A, hypochromic properties are achieved in aerobic conditions when using Vc as a sacrifice against the air oxidation. To confirm that the hypochromic properties originate from the oxidation of [Mo_132_] and to accelerate the process, 365 nm irradiation was then performed in parallel with and without Vc in buffer A (Figure S2B). After 12 h, 20% of the absorbance was diminished for the latter case; however, almost no change was observed for the former.

**Interaction of capsid protein for colorimetry change of [Mo_132_].** The non-covalent interactions of POMs with several types of biomolecules have been investigated extensively over the past years [35,36,37]. Based on the structural features of POMs, it is evident that the negatively charged [Mo_132_] mainly provide electrostatic interactions with a variety of cationic species of protein. However, the observed hypochromic effect here should be essentially conducted by the external oxidation sourced from the protein rather than the buffer solution or the aerobic environment, since the color degradation does not fully occur in such a short time. Several peptides and proteins with specific surface charges have been used to examine the hypochromic effect of [Mo_132_] (Figure S3). Under neutral conditions, the negative peptide of pTau-aac (pI = 4.5) and protein of BSA (pI = 4.6) do not induce a significant absorption change of [Mo_132_]; however, the positively charged ones of dTau30 (pI = 10.4) and lysozyme (pI = 11.0) drive a significant decrease in absorption, indicating the vital role of positive partners to the [Mo_132_] hypochromic effect. We can infer that the exposed basic residues in L1 accelerate the oxidation of [Mo_132_].

The cryo-electron microscopy and image analysis on capsid proteins [37] revealed that the L1-p bind together to form VLPs via a long segment of the C-terminal (Figure S4A). The images revealed that the sequence after Asp401 at the C-terminal extended from one pentamer to the adjacent one to strengthen the VLPs structure. This segment was dominated by cationic residues such as arginine or lysine (Figure S4B), which are firmly prone to bind with the negatively charged [Mo_132_]. After L1-p assembles into VLP, however, this segment is embedded in the wall of the VLP sphere, and the charged environment varies widely (Figure S4C). To further confirm the binding site of [Mo_132_] with capsid protein, the sequence of two peptides, pep1–401 and pep401–495, were constructed and expressed separately, through identical approaches as the full-length L1.

**Figure 7:**
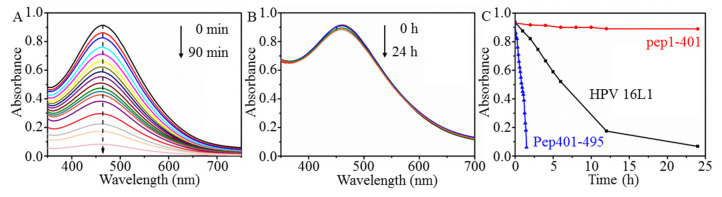
Time-dependent UV–Vis absorption spectra of [Mo_132_] (2.5 µM) in buffer A in the presence of (**A**) pep401–495 (10.0 µM) and (**B**) pep1–401(10.0 µM), respectively. (**C**) The plot of the corresponding intensity changed at 456 nm in (**A**,**B**), and the mixture of [Mo_132_] and L1-p in buffer A, respectively, vs. time for comparison.

After carrying out DNA sequence assays (Figure S5) and protein purification, each peptide was mixed with [Mo_132_] in buffer A. This procedure yielded different phenomena. A quick decrease in [Mo_132_] absorption at 456 nm was shown when mixed with pep401–495 (Figure 7A). The band disappeared completely, and the solution became pale within 90 min. The time-dependent plot of absorption (Figure 7C) revealed that the peptide drives the hypochromic effect of the cluster much faster than that of L1-p (Figure 1A), confirming a decisive role of pep401–495 in this process. In contrast, mixing pep1–401 with [Mo_132_] only led to a very slight decrease in absorption centered at 456 nm (Figure 7B), indicating its feeble contribution in L1 to the [Mo_132_] hypochromic effect. The plots of absorption intensity vs. time (Figure 7C) clearly illustrate the responsive differences between pep401–495, pep1–401, and L1-p to [Mo_132_], further demonstrating that the binding with basic residues of pep401–495 causes the enhanced hypochromic effect by L1. Thus, it can be speculated that during the assembly of L1-p to VLP, the stronger binding affinity between L1-p subunits could force [Mo_132_] to be released from the positive sites of L1-p and electrostatically attach to other positions of VLPs. Considering the positive areas at the interior surface of VLPs [38], the negatively charged [Mo_132_] are easy to adsorb on the inner surface of VLP, which shield [Mo_132_] from oxidation and consequently protect it from the hypochromic effect. Moreover, the observed larger aggregates of VLPs induced by [Mo_132_] (Figure 5 and Figure 6) suggest an additional protection of [Mo_132_].

**Figure 8:**
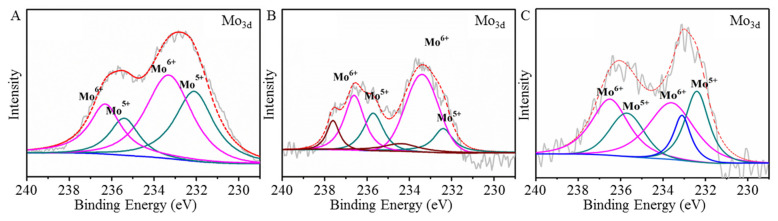
XPS of (**A**) [Mo_132_] in buffer A; (**B**) [Mo_132_] and L1-p in buffer A; (**C**) [Mo_132_] and VLPs in the assembly buffer after incubation at the aerobic condition for 24 h.

Redox nature of [Mo_132_] in the presence of L1-p and VLP. X-ray photoelectron spectroscopy (XPS) was used to analyze the redox state of pristine [Mo_132_] (Figure 8A) and in the presence of L1-p (Figure 8B) or VLP (Figure 8C) under aerobic conditions. The characteristic Mo_3d_ doublet, composed of the 3d_5/2_ and 3d_3/2_ levels resulting from spin-orbit coupling, was observed in the spectra. Suitable fits of the data points, corresponding to two possible 3d doublets of Mo in different oxidation states of Mo(VI) and Mo(V), were achieved using two pairs of Lorentzian–Gaussian functions. The peaks centered at 232.4 and 235.5 eV were assigned to Mo(V), while those at 233.6 and 236.6 eV were attributed to Mo(VI), respectively [39,40]. Semi-quantitative calculations showed that the characteristic ratio of peak area for Mo(VI) and Mo(V) was 1.45:1 for [Mo_132_] (Table 1), which was slightly over the value of 1.2 calculated from the ratio of 72 Mo(VI) to 60 Mo(V). The reason for this deviation can be deduced from the aerobic oxidation of partial Mo(V) atoms (approximate 10) in [Mo_132_] solution during sample preparation.

The fitting curves of [Mo_132_] in mixing with L1-p (Figure 8B) were vastly different from [Mo_132_] alone. Besides the binding energy pairs attributed to Mo(V) and Mo(VI), the third coupled binding energy bands were observed. The latter were attributed to the intermediate of Mo during the transition. Furthermore, the quantitative calculation revealed that the ratio of peak area for Mo(VI) and Mo(V) increased to 2.5:1 (Table 1), indicating that more Mo(V) atoms had been oxidized. Larger amounts of Mo(V) (approximate 31) in [Mo_132_] were oxidized into Mo(VI) after binding with L1-p, which is solid evidence for the enhanced hypochromic effect in the mixture.

**Scheme 2:**
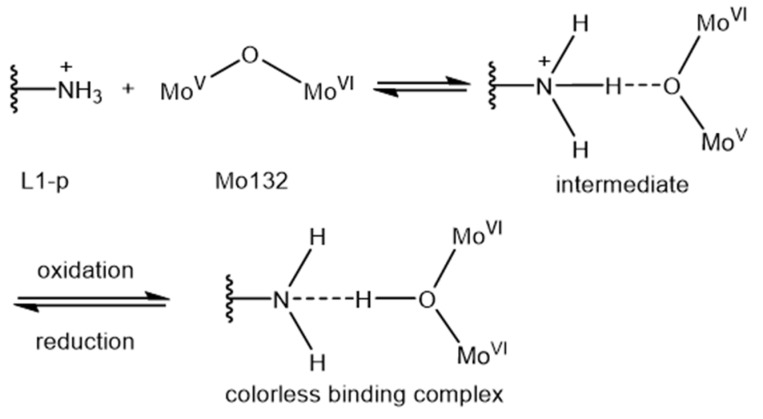
Schematic drawing of the chromic mechanism of [Mo_132_] induced by protein.

Accompanying the assembly of L1-p, the XPS results of [Mo_132_] almost returned back to the state of [Mo_132_] alone in solution (Figure 8C). Although the intermediate component of Mo atoms still appeared in the fitting model, the calculation revealed a reduction in band ratio for Mo(VI) to Mo(V) of approximately 1.45:1 (Table 1). Seventeen of the oxidized Mo(VI) were reduced back to Mo(V). As a result, the L1-p presented here is similar to a sensitizer for [Mo_132_] hypochromic properties. The electrostatic interaction between [Mo_132_] and pep401–495 connecting the positive residue and [Mo_132_] directly allows for the oxidation of Mo(V) to Mo(VI) more easily. Consequently, the intervalence charge transfer between Mo(V) and Mo(VI) centers was vastly weakened, and the color finally disappeared with time. As illustrated, such a process is accompanied by the binding with free basic residues in protein (Scheme 2) and the elimination of them in L1-p after assembly into VLP (Figure S4C).

## 3. Conclusions and Perspectives

We report the opposite color response of a giant polyoxometalate, (NH_4_)_42_[Mo_132_O_372_(CHCOO)_30_] (H_2_O)_72_ ([Mo_132_]), to the existing states of human papillomavirus (HPV) major capsid protein, L1-pentamer (L1-p) and virus-like particles (VLPs), originating from the assembly between [Mo_132_] and capsid protein. Assembly with L1-p resulted in the improved hypochromic of [Mo_132_] while assembly with the as-assembled VLPs led to an obvious protection of [Mo_132_] from the hypochromic effect, compared to the single cluster in solution. Furthermore, both the size and morphology of the assemblies were characterized by using CsCl gradient centrifugation, SDS-PAGE, DLS, and TEM imaging. Remarkably, the in-depth mechanism studies determined by XPS and two well-designed peptides from L1 disclose that the electrostatic interaction between [Mo_132_] and pep401–495 induces the change of Mo(V) into Mo (VI), which facilitates the hypochromic effect of [Mo_132_]; however, the assembly from L1-p to VLPs reduced the binding possibility of [Mo_132_] to pep401–495 and the transition from Mo(V) to Mo (VI), which protects [Mo_132_] from the hypochromic effect. Therefore, the present study not only reports a unique phenomenon of contrasting color responses of [Mo_132_] to HPV capsid protein, 16 L1-p and VLPs (which affords an easily performed colorimetry approach to evaluate the states of the HPV capsid protein), but also extends the potential of the molybdate polyoxometalate family toward new applications in medical science and could possibly be extended to other kinds of POMs.

## 4. Experimental Section

### 4.1. Reagents and Materials

Tryptone was purchased from OXOID Ltd. (Basingstoke, UK). Isopropyl-β-d-thiogalactoside (IPTG) and kanamycin were acquired from Tianjia Tech, China. Ethylene diamine tetraacetic acid (EDTA), acetic acid, ethanol, and methanol were obtained from BCIGC Ltd., China. Coomassie blue G-250 and Tween 80 were produced from DingGuo Ltd. (Beijing, China). 3-(*N*-morpholinyl) propanesulfonic acids (MOPs), and NaCl used for buffer preparation were purchased from Aladdin, China. D,L-dithiothreitol (DTT) and Tris(hydroxymethyl)aminomethane (Tris) are products from the Coolaber Company in China. The purity of all chemicals was higher than 99.9% and used as received.

### 4.2. Expression, Purification of HPV 16 L1 Protein and Peptides

The coding sequences and preparation of major capsid protein (L1) for HPV 16 followed the procedures described in a previous work where pET-30a vector and BL21 Star^TM^ (DE3) were employed for better expression [40]. Briefly, the recombinant *Escherichia coli* strain BL21 Star^TM^ (DE3) was cultured at 37 °C with stirring at 220 rpm, where the bacteria were induced by 0.1 mM IPTG. When OD_600_ reached 0.6–0.8, the cells were further cultivated at 25 °C for another 17 h, and then they were harvested by centrifugation. The cell pellets were re-suspended in buffer A (50 mM MOPs, 250 mM NaCl, 10 mM DTT, pH 7.0) with a concentration of 1.0 g in 10 mL. After being lysed by sonication and separated by centrifugation, a supernatant rich with L1 protein was obtained and further stabilized for another 30 min with increased DTT (20 mM) in buffer A. Then, purification was performed again by using a cationic exchange column filled with POROS^®^ XS (xk16/10), where the supernatant was introduced into the column after being equilibrated with buffer A. A solution of 1.2 M NaCl finally eluted the target protein L1, and its final concentration was determined using the BCA method. The purity of L1 was finally identified by using 12% (wt/vol) SDS-PAGE. The obtained HPV 16 L1 existed essentially in the form of VLPs, which was further dialyzed against disassembly buffer (50 mM tris, 100 mM NaCl, 2 mM EDTA, 20 mM DTT, 0.01% Tween 80, pH 8.0) at 4 °C for 24 h (being changed every 4 h) to disassemble VLPs into L1-p for co-assembly monitoring.

The coding sequences of HPV 16 pep1–401 and pep401–495 were the same as the corresponding segment of HPV 16 L1 [40]. The pET-30a vector containing the respect gene was transformed into *Escherichia coli* strain BL21 Star^TM^ (DE3) and cultured at 37 °C. The experimental conditions and method used for their expression and the following purifications were the same as that of the HPV 16 L1 protein described above.

### 4.3. Preparation of (NH_4_)_42_[Mo_132_O_372_(CH_3_COO)_30_]∙72H_2_O

The synthesis and structural characterization of the [Mo_132_] cluster followed procedures similar to those reported elsewhere [18]. Briefly, 0.8 g N_2_H_4_·H_2_SO_4_ was added into 250 mL of H_2_O containing 5.6 g of (NH_4_)[Mo_7_O_24_]·4H_2_O and 12.5 g of CH_3_COONH_4_. After stirring for 10 min, 83 mL of 50% CH_3_COOH (*v*/*v*) was added to replace the involved –SO_4_^2−^, further stabilizing and giving the product a crystalline precipitate. After desiccation in air, the product was characterized by X-ray diffraction, IR, and elemental analysis. The 100 μM of stock solution of [Mo_132_] was then prepared in deionized water, which was further diluted to the desired concentration when ready for use.

### 4.4. Assembly/Disassembly Monitoring of Mo_132_ and HPV 16 L1

Four typical samples were prepared, respectively, for the response assay of [Mo_132_] to the HPV capsid protein: (I) the stock solution of [Mo_132_] was mixed with L1-p and stirred at 4 °C for 24 h in buffer A to obtain full binding, where the concentration of inorganic clusters is 2.5 μM and that of proteins is 10 μM; (II) the mixture of (I) was subsequently dialyzed in a 1.0 L assembly buffer (10 mM phosphate, 500 mM NaCl, 0.03% Tween 80, pH 5.4) at 4 °C for 24 h to induce the assembly of L1-p into VLPs in the presence of [Mo_132_]; (III) 10 μM of L1-p was dialyzed in 1.0 L assembly buffer to obtain full-sized empty VLPs, which was used either as a control or further mixed with [Mo_132_] for post-assembly monitoring as the number (IV) sample, after another 24 h stirring at 4 °C.

In a parallel experiment, sample (II) was further dialyzed against 1.0 L of disassembly buffer (50 mM Tris, 100 mM NaCl, 2 mM EDTA, 20 mM DTT, 0.01% Tween 80) at 4 °C for 24 h to record the disassembly behavior of VLPs into L1-p in the presence of [Mo_132_]. The concentration of [Mo_132_] used in all of the above experiments was the same at 2.5 μM unless mentioned otherwise, while that for HPV 16 L1 was 10 μM, depending on the L1 monomer. In addition, the procedures used to monitor the conversion between L1-p and VLPs were the same as reported previously [27,28].

### 4.5. Cesium Chloride Gradient Centrifugation

First, we prepared the discontinuous densities of CsCl aqueous solution at 1.50, 1.35, and 1.25 g/mL, respectively. Then, the solutions in a volume of 0.5, 3.0, and 3.5 mL were slowly added into ultracentrifuge tubes in order, respectively. After further adding the mixture solution of the L1-p protein and Mo_132_ with equivalent volume to the upper part separately, the ultracentrifuge tubes were placed in the SW40 rotor and centrifuged at 220,000× *g* for 3.5 h at 4 °C. Finally, the interest bands and the counterparts were collected using a needle and dialyzed in PBS buffer for 24 h to remove the attached CsCl. The products were identified by 12% SDS-PAGE, dynamic light scattering (DLS), and transmission electron microscopy (TEM).

### 4.6. Instruments

UV–Vis absorption spectra were recorded on a Shimadzu (Kyoto, Japan) RF-5301PC spectrometer for the assembly and/or disassembly of HPV 16 L1 protein or peptides. A 1.0 mL target solution was added in a quartz cuvette, and the spectra in the 300–700 nm range were collected for each sample. The particle size was assayed by DLS. Briefly, after pre-filtration performed by placing the sample in a PCS1115 cuvette, the particle sizes were tested on a Malvern Zetasizer Nano-ZS 90 (Malvern, England) at 25 °C. All data were repeated for each sample in three parallel sets. The procedures in the sample preparation for TEM imaging were the same as those described previously [21,22]. The samples without staining were first spotted on copper grids coated with carbon and formvar. After drying in the air for 2 min, the measurements were carried out on a H-7650 transmission electron microscope (Hitachi, Tokyo, Japan) under an accelerating voltage of 80 kV. The images of samples with a stain of 2% phosphotungstate were collected under an accelerating voltage of 120 kV. The X-ray photoelectron spectroscopy (XPS) measurements were acquired using Thermo ESCALAB 250, where an Al K_α_ line (1486.6 eV) was employed as a monochromic X-ray source and the binding energy of C1s (284.6 eV) was used for correction.

## Figures and Tables

**Table 1:** Integrated area of the simulated peak for Mo(V) and Mo(VI) from the XPS results in Figure 8A–C, respectively, and their ratio.

Samples	[Mo_132_]	[Mo_132_] + L1-p	[Mo_132_] + VLPs
Mo(V)	115,359.60 + 43,910.59	15,696.10 + 18,827.48	17,349.15 + 21,444.59
Mo(VI)	147,757.50 + 82,513.98	58,321.11 + 28,475.33	26,737.09 + 32,049.94
Ratio of Mo(VI) to Mo(V)	1.45:1	2.5:1	1.5:1

## Supplementary Materials

The following are available online at https://www.mdpi.com/article/10.3390/nano12050736/s1, Figure S1: (A) Time-dependent UV-vis absorption spectra of [Mo_132_]@VLPs in disassembly buffer, to induce the disassembly of VLPs into L1-p again; (B) The plots of corresponding intensity in (A), which show obvious hypochromicity after 3 h incubation. Finally, more that 90% color was diminished again; Figure S2: Time-dependent UV-vis absorption spectra of [Mo_132_] in buffer A (2.5 µM) (A) in the presence of Vc (10.0 µM) under white light; (B) in the absence and presence of Vc (10.0 µM) under the irradiation of white light or 365 nm, respectively; Figure S3: Time-dependent UV-vis absorption spectra of [Mo_132_] in buffer A (2.5 µM) in the presence of the negative (A) peptide, pTau-aac (10.0 µM, pI = 4.5), (B) protein, BSA (10.0 µM, pI = 4.6); and positive (C) peptide, dTau30 (10.0 µM, pI = 10.4), (D) protein, lysozyme (10.0 µM, pI = 11.0), respectively; Figure S4: Illustration of 3D structural relationship between one HPV16 L1-p and its neighbor. The model is obtained from a Cryo-EM reconstruction structure (PDB ID, 3J6R).^S1^ (A) Top view on L1-p; (B) the enlarge part in a box of (A), highlighting the involved arginine and lysine, respectively; (C) Side view of L1-p to show more clearly the burier of pep401–495 segment by the neighboring L1-p in VLP; Figure S5: Agarose gel electrophoresis to assay the DNA sequence of two peptides derived from HPV 16L1. (A) Enzyme digestion of T-easy-peptide 401–495. The gene of peptide 401–495 was 285 bp, corresponding to the site between 250 to 500 bp in marker. (B) Enzyme digestion of T-easy-peptide 1–401. The gene of peptide 1–401 is 1203 bp, corresponding to the site between 1000 to 1500 bp in marker, Table S1: Integrated area of the simulated peak for Mo(V) and Mo(VI) from the XPS results in Figure 8A,B, and C, respectively, and the ratio of them.
