# Supplementary material for: A Visual Discrimination of Existing States of Virus Capsid Protein by a Giant Molybdate Cluster
Source: Nanomaterials (Basel). 2022 Feb 22;12(5):736. doi: 10.3390/nano12050736 (PMC8911902; doi:10.3390/nano12050736)
Supplement: Supplementary file 1 [file nanomaterials-12-00736-s001.zip › nanomaterials-1576667-supplementary.pdf]

nanomaterials

Supplementary Material

A Visual Discrimination of Existing States of Virus Capsid Protein by a Giant Molybdate Cluster

Yarong Xue [1], Mingfen Wei [1], Dingyi Fu [3], Yuqing Wu [1,*], Bo Sun [2], Xianghui Yu [2] and Lixin Wu [1,*]

[1] State Key Laboratory of Supramolecular Structure and Materials, Institute of Theoretical Chemistry, Jilin University, No.2699 Qianjin Street, Changchun 130012, China; xueyr17@mails.jlu.edu.cn (Y.X.); weimf19@mails.jlu.edu.cn (M.W.)

[2] State Engineering Laboratory of AIDS Vaccine, Jilin University, No.2699 Qianjin Street, Changchun 130012, China; bo_sun@jlu.edu.cn (B.S.); xianghui@jlu.edu.cn (X.Y.)

[3] School of Pharmacy Nantong University, No. 19 Qixiu Road, Nantong 226001, China; fudingyi@ntu.edu.cn

* Correspondence: yqwu@jlu.edu.cn (Y.W.); wulx@jlu.edu.cn (L.W.)

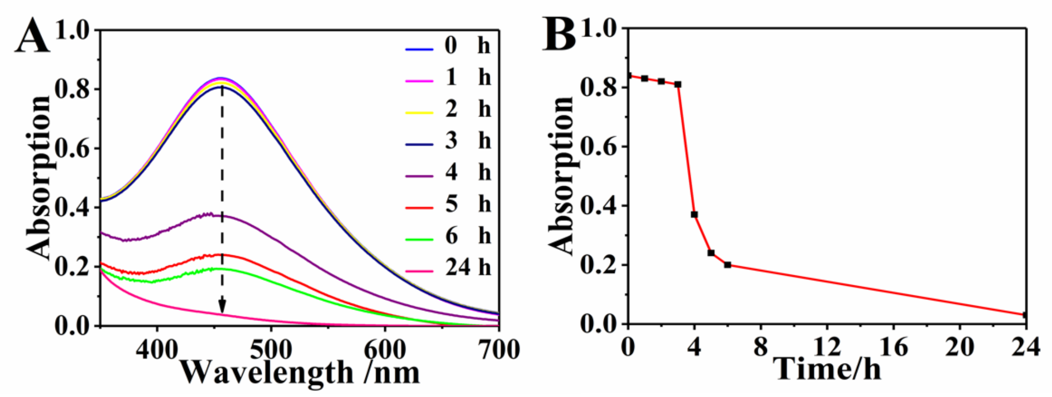

Figure S1. (**A**) Time-dependent UV-vis absorption spectra of $[Mo_{132}]$@VLPs in disassembly buffer, to induce the disassembly of VLPs into L1-p again; (**B**) The plots of corresponding intensity in (A), which show obvious hypochromicity after 3 h incubation. Finally, more that 90% color was diminished again.

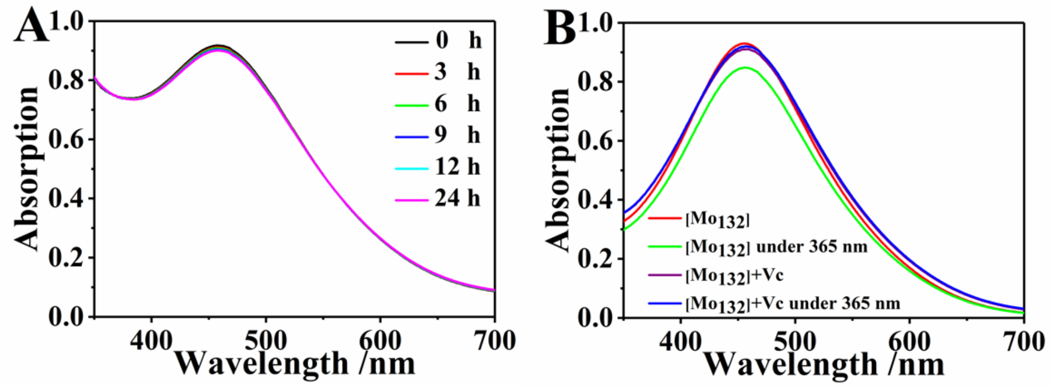

Figure S2. Time-dependent UV-vis absorption spectra of $[Mo_{132}]$ in buffer A (2.5 μM) (**A**) in the presence of Vc (10.0 μM) under white light; (**B**) in the absence and presence of Vc (10.0 μM) under the irradiation of white light or 365 nm, respectively.

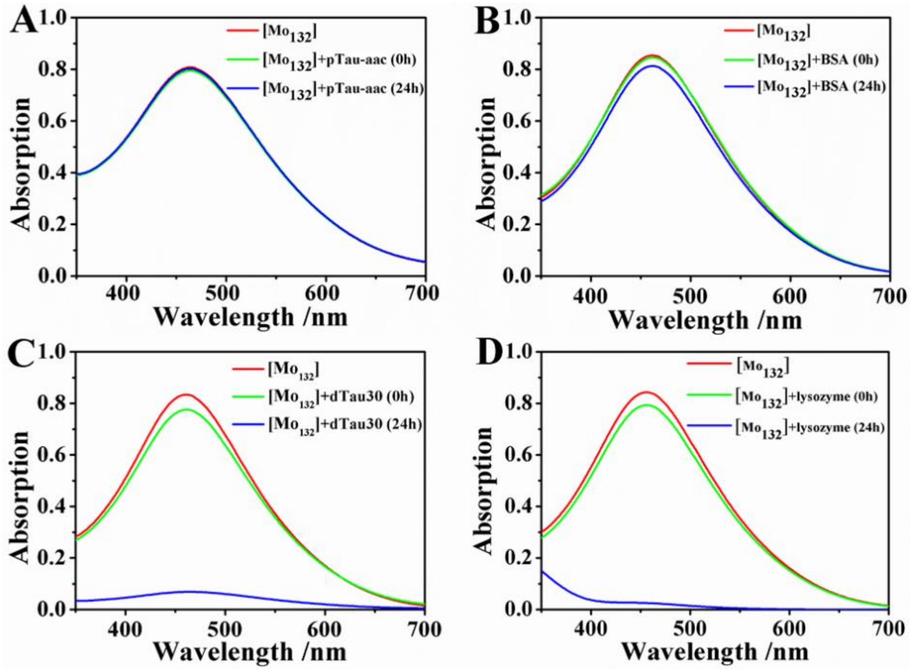

Figure S3. Time-dependent UV-vis absorption spectra of [Mo$_{132}$] in buffer A (2.5 μM) in the presence of the negative (**A**) peptide, pTau-aac (10.0 μM, pI = 4.5), (**B**) protein, BSA (10.0 μM, pI = 4.6); and positive (**C**) peptide, dTau30 (10.0 μM, pI = 10.4), (**D**) protein, lysozyme (10.0 μM, pI = 11.0), respectively.

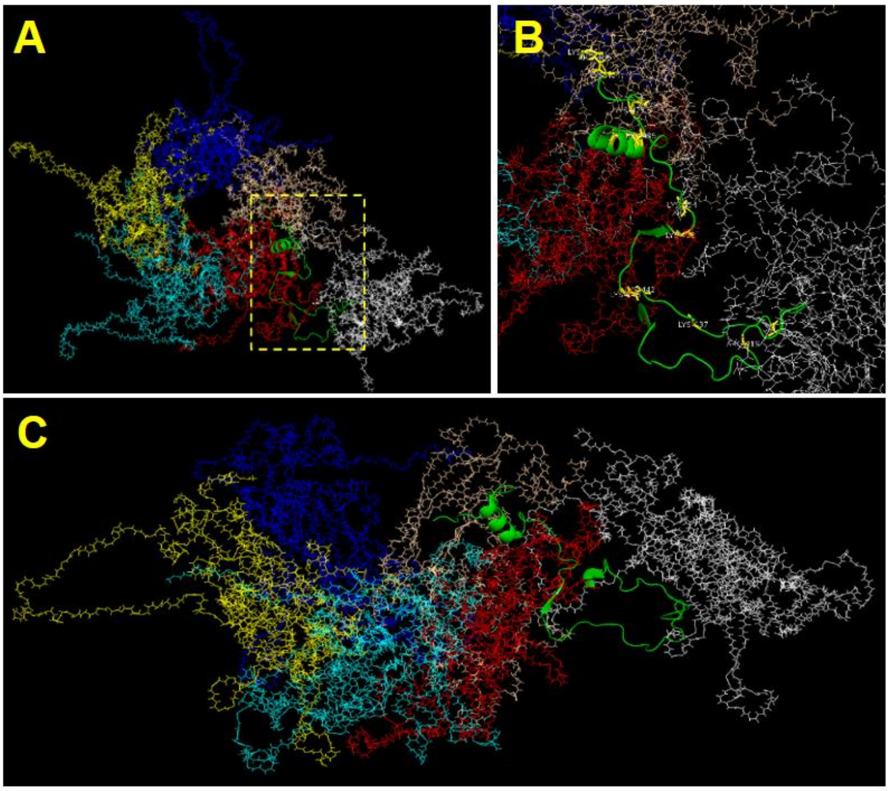

Figure S4. Illustration of 3D structural relationship between one HPV16 L1-p and its neighbor. The model is obtained from a Cryo-EM reconstruction structure (PDB ID, 3J6R).[S1] (**A**) Top view on L1-p; (**B**) the enlarge part in a box of (**A**), highlighting the involved arginine and lysine, respectively; (**C**) Side view of L1-p to show more clearly the burier of pep401–495 segment by the neighboring L1-p in VLP.

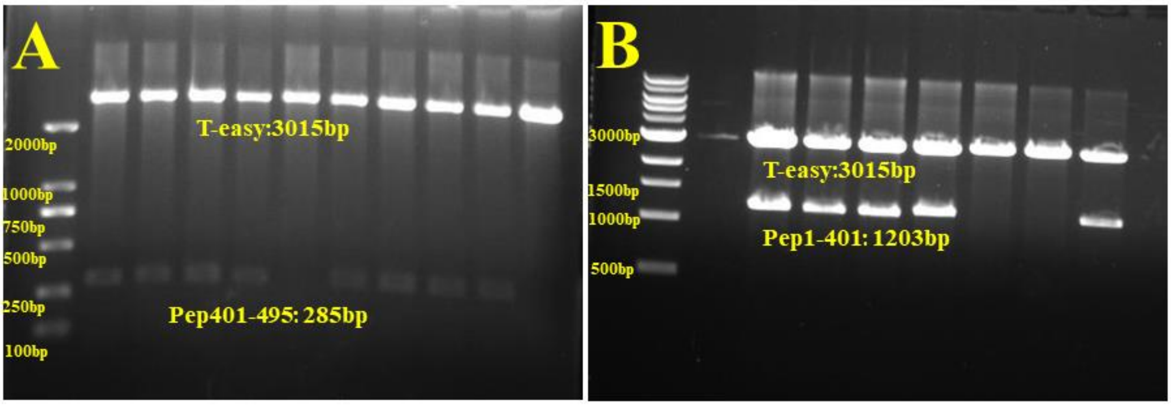

Figure S5. Agarose gel electrophoresis to assay the DNA sequence of two peptides derived from HPV 16L1. (**A**) Enzyme digestion of T-easy-peptide 401–495. The gene of peptide 401–495 was 285 bp, corresponding to the site between 250 to 500 bp in marker. (**B**) Enzyme digestion of T-easy-peptide 1–401. The gene of peptide 1–401 is 1203 bp, corresponding to the site between 1000 to 1500 bp in marker.

Table S1. Integrated area of the simulated peak for Mo(V) and Mo(VI) from the XPS results in Figure 8A,B, and C, respectively, and the ratio of them.

	**[Mo_{132}]**	**[Mo_{132}] + L1-p**	**[Mo_{132}] + VLPs**
Mo(V)	115359.60+43910.59	15696.10+18827.48	17349.15+21444.59
Mo(VI)	147757.50+82513.98	58321.11+28475.33	26737.09+32049.94
Area ratio Mo(VI) to Mo(V)	1.45 : 1	2.5 : 1	1.5 : 1

References:

1. Cardone, G.; Moyer, A.L.; Cheng, N.; Thompson, C.D.; Dvoretzky, I.; Lowy, D. R.; Schiller, J.T.; Steven, A.C.; Buck, C.B.; Trus, B.L. Maturation of the Human Papillomavirus 16 Capsid. *mBio* **2014**, *5(4)*, e01104–e01114.
